# Clinicopathological and Immunohistochemistry Study of a Long Survivor of Giant Cell Glioblastoma in a Patient With Neurofibromatosis 1: Case Report

**DOI:** 10.7759/cureus.39014

**Published:** 2023-05-14

**Authors:** Noora Al-romaihi, Mohammed Awadh, Mohammed Albalooshi, Mohammed Ahmed, Abdulla Darwish

**Affiliations:** 1 Pathology and Laboratory Medicine, Bahrain Defense Force Hospital Royal Medical Services, Riffa, BHR; 2 Neurological Surgery, Bahrain Defense Force Hospital Royal Medical Services, Riffa, BHR; 3 Radiology, Bahrain Defense Force Hospital Royal Medical Services, Riffa, BHR

**Keywords:** glioblastoma therapy, glioblastoma idh-wildtype, genetics of glioblastoma, glioma types, glioblastoma survivor, prognosis, giant cell glioblastoma, glioblastoma multiforme

## Abstract

Glioblastoma multiforme (IDH wild type) is an aggressive glial tumor of astrocytic origin (WHO-grade 4) with a two-year median survival period. Patients who live more than three years are considered as long survivors. In this study, we present a long survivor of a known case of neurofibromatosis type 1 who developed GBM of the giant cell type at age 14 years, and now the patient, at age 28, has been cancer-free for more than 14 years.

## Introduction

Neurofibromatosis type 1 (NF1) is an inherited neurocutaneous disease characterized by skin pigmentation (café-au-lait spots) and nerve tumors that affect a variety of systems in the body. The presence of two out of seven distinct features will be diagnostic for NF1, such as café au lait spots, freckling, Lisch nodules, plexiform neurofibromas, bony lesions, optic gliomas, and family history, as per the National Institute of Health Consensus Development Conference in 1988 [1]. The approximate incidence of NF1 is around 1:2600 to 1:3000. The peripheral and central nervous systems can be involved in NF1. Brain involvement is manifested by the development of glial tumors, including low- and high-grade gliomas. Neurofibromatosis type 1 (NF1) increases the chance of developing central nervous system (CNS) tumors [2]. Furthermore, CNS tumors are the second most common tumors in children [3]. Glioblastoma multiforme (GBM)-wild type, which lacks mutations in isocitrate dehydrogenases (IDH1) and (IDH2), is a high-grade glioma with a devastating outcome, and it is known as having one of the worst five-year survival rates in all malignancies [4-5]. In this study, we present a long survivor of a known case (NF1) who developed GBM of the giant cell type at age 14 years, and now the patient, at age 28, has been cancer-free for more than 14 years, still alive and in good condition.

## Case presentation

**Figure 1:**
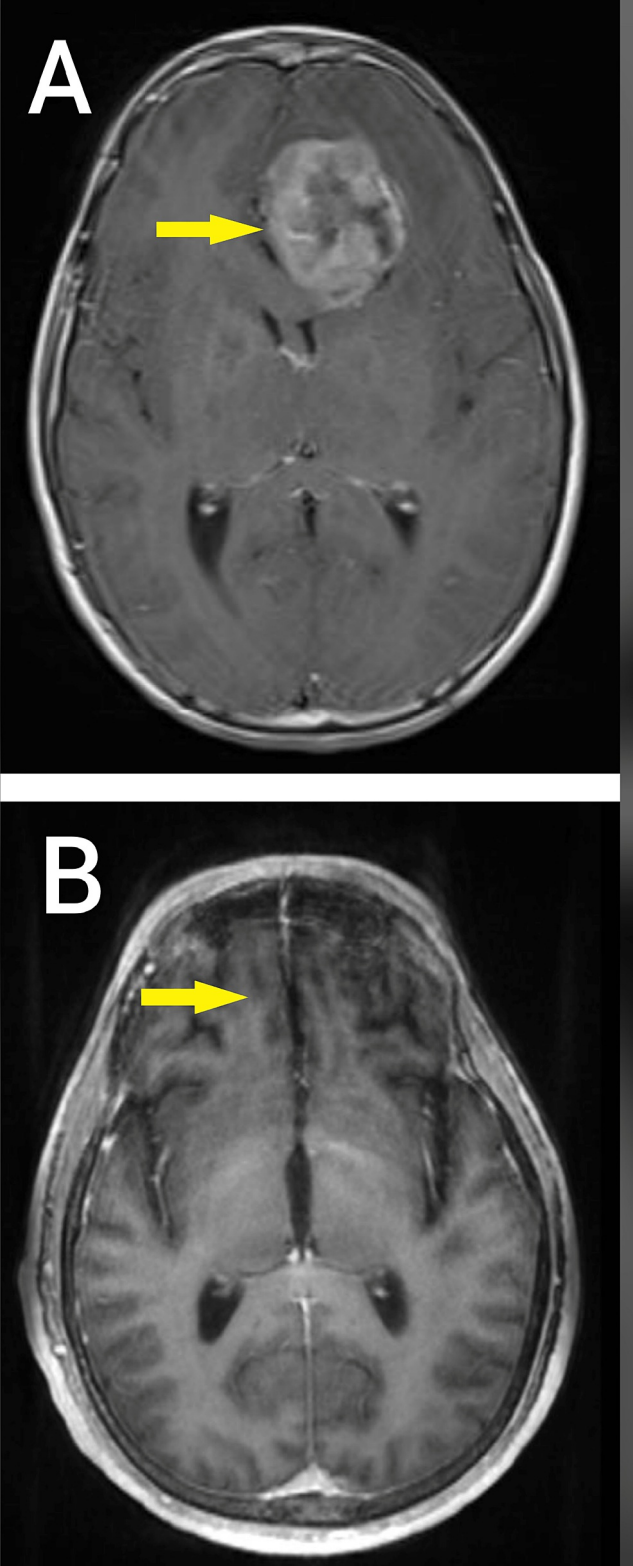
T1-weighted MRI images: (A) Pre-operative MRI dated on 6.7.2008 revealed evidence of a large left frontal intra-axial mass lesion, shown by the arrow arising medially and extending towards the genu of the corpus callosum, associated with irregular enhancement. There is a mass effect with displacement of the midline to the right side and effacement of the anterior horn of the left lateral ventricle. (B) MRI dated on 11.10.2021, revealed evidence of post-surgical changes in the form of mild gliosis shown by the arrow; otherwise, no mass lesion is shown.

**Figure 2:**
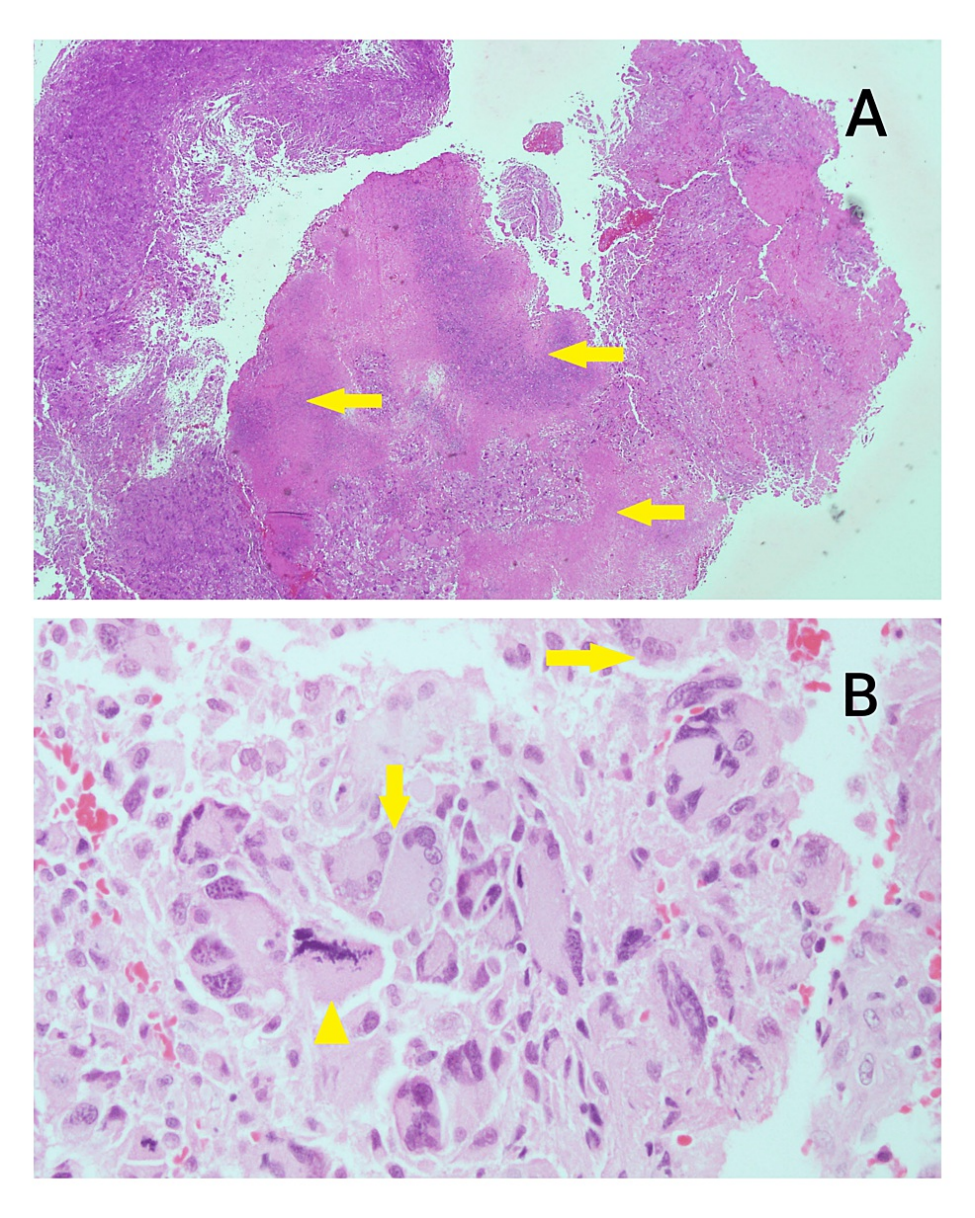
Microscopic view of the glioblastoma multiforme A: shows hypercellular tumors with large areas of geographic necrosis (pinkish and bluish areas, marked by arrows); B: shows highly atypical pleomorphic tumor cells, including multinucleated forms (marked by arrows) with many atypical mitotic figures, marked by an arrowhead (H&E stain).

**Figure 3:**
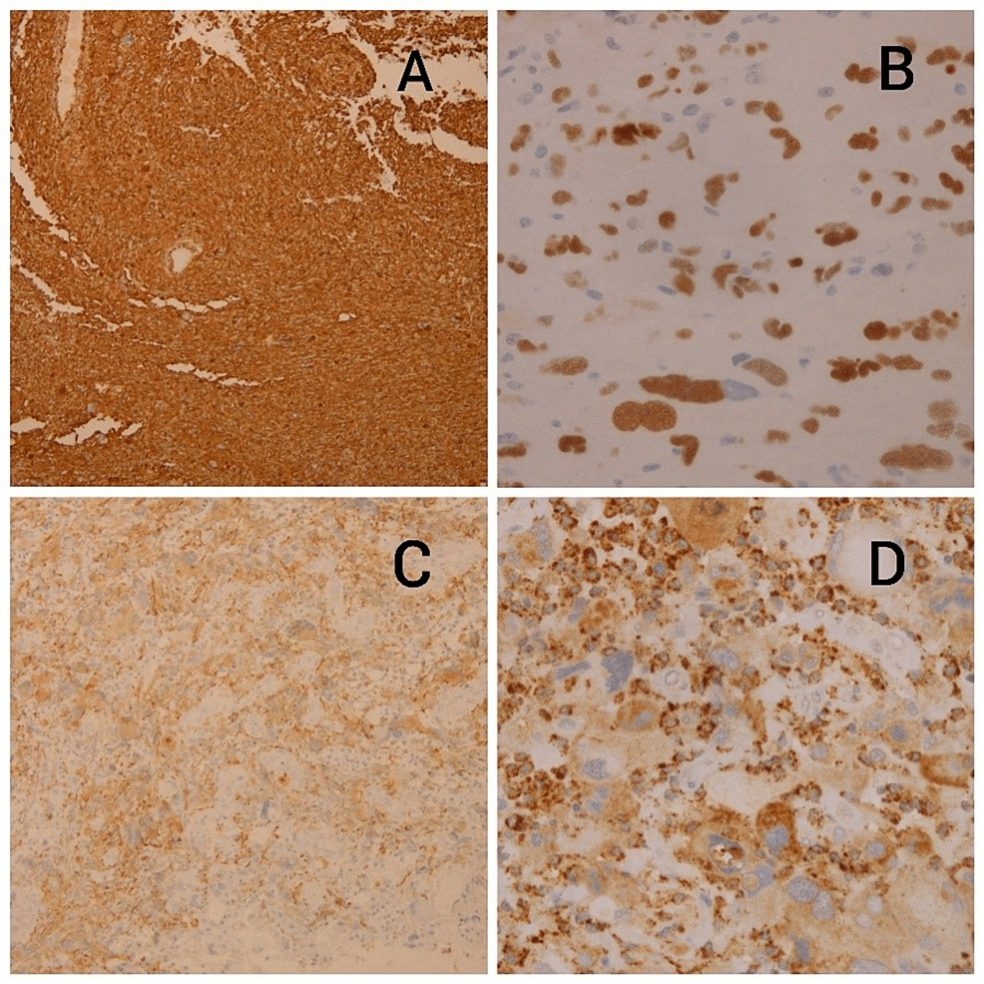
The immunohistochemistry study of the tumor: A: strong reaction for Vimentin, B: overexpression for P53, C: positive for GFAP, D: positive for the CD68 marker.

A 14-year-old male known case of NF1 manifested clinically by multiple cafe au lait spots, multiple skin nodules (neurofibromas), and delayed maturation. The patient was diagnosed with giant cell glioblastoma grade IV in 2008. He came with a history of persistent vomiting, headaches, and photophobia for three weeks with no other neurologic symptoms. He had a CT brain scan followed by an MRI, which revealed a large left frontal intra-axial lesion arising medially and extending posteriorly toward the genu of the corpus callosum (Figure 1A). It was well-circumscribed and enhanced homogeneously for contrast. The picture was more consistent with high-grade glioma. On July 8, 2008, he underwent a left frontal craniotomy, and gross total resection of the tumor has been achieved. A frozen section was submitted as three fragments of soft grayish-pink brain tissue ranging in size from 1x0.5x0.3 cm to 0.8x0.5x0.2 cm (x1) grossly. The frozen section diagnosis was in keeping with a malignant pleomorphic tumor consistent with a grade IV giant cell glioblastoma. The final pathology report was in concordance with the frozen section diagnosis of giant cell glioblastoma (wild type) grade IV. The tumor showed an infiltrative pattern of growth with nuclear atypia, high mitotic activity, and microvascular proliferation with extensive necrosis. There were many markedly pleomorphic, bizarre cells, including multinucleated giant cells (Figures 2A, 2B). An immunochemistry study showed the tumor cells were positive for vimentin, GFAP (patchy positive), S100 (weak positive), overexpression of P53, and CD68 (Figures 3A-3D). The Ki67 proliferation index of tumor cells is 70%. The tumor was negative for IDH and EGFR immunomarkers.

Clinically, the patient is well and free of symptoms. After that, the patient was referred to an oncology center and received craniospinal radiation and concurrent chemotherapy with temozolomide for a total of six weeks and a total of 10 cycles with temozolomide (200 mg/m2/day x 5 days per cycle) as maintenance. He responded well to the treatment. On February 9, 2009, he had a partial seizure which was managed with carbamazepine 200 mg. Since then, he has been well and stable and has had 3-4 episodes. The patient has been off treatment since July 2009, and he is undergoing MRI brain and spinal studies every three months. The latest MRI of the brain was on October 11, 2021, and revealed evidence of post-surgical changes in the form of mild gliosis; otherwise, no mass lesion was shown (Figure 1B).

## Discussion

Glioblastoma multiforme (IDH wildtype) is an aggressive glial tumor of astrocytic origin. The tumor is characterized by cellular pleomorphism, nuclear atypia, many mitoses, vascular proliferation, and tumor necrosis (WHO grade IV). Morphologically, GBM has a different microscopic pattern, predominantly astrocytic differentiation. Other patterns, such as small cells, sarcomatous, multinucleated giant cells, and gemistocytic types, are described [6-8]. The male-to-female ratio is 1.6:1, with a two-year median survival period. Patients who live more than three years are considered long survivors [8]. Giant cell glioblastoma is a subtype of glioblastoma that occurs more commonly in children with a better prognosis.

Many studies revealed that young age, female gender, easily resection tumors, the presence of abundant bizarre multinucleated giant cells, a Ki-67 proliferation index of less than 18%, and multimodality management are associated with a longer survival period [3,5,9,10]. Despite the fact that Huttner et al. and Krex et al. mentioned in their studies that multimodality management will not affect the median survival period [2,5], on the other hand, the presence of p53 and Ki-67 proliferation indexes greater than 36% indicates a bad prognosis [2,3]. However, although our patient had a positive p53 and Ki-67 proliferation index of 70%, he showed a better outcome. According to Romo et al., metastasis is rare in GBM, but it may appear at the age of 40 [9]. Hargrave and Zacharoulis illustrated in their study a comparison between two groups, one group that received radiotherapy and chemotherapy and the other group that received radiotherapy alone; their median survival was 14.6 months and 12.1 months, respectively, and their two-year survival rates were 26.5% and 10.4%, respectively [3]. Our study suggests that proper patient management, including complete tumor resection, temozolomide intake, and radiotherapy, may have a better outcome. Despite the fact that Huttner et al. and Krex et al. mentioned in their studies that multimodality management will not affect overall survival at two years, the rating is between 10% and 30%, and the prognosis is poor [2,5].

Genetic disorders such as tuberous sclerosis, Turcot syndrome, multiple endocrine neoplasia type IIA, and NFI may play a role in the pathogenesis of GBM, which is the case in our patient [10-12]. According to many studies, a mutation in the NF1 gene, which acts as a regulator of cell growth, can lead to tumor formation in astrocytomas, including glioblastomas, but if it is associated with a P53 mutation, these tumors may show a good prognosis [1,2,10,13]. As mentioned previously, positive p53 alone can result in a bad prognosis, but if it is associated with NF1, it will result in a better outcome. In addition, Huttner et al. stated that the two-year survival of patients with NF1-associated glioblastoma is 60% compared to 25% in non-NF1 patients [2]. In a study carried out by Huttner et al., it was concluded that NF1 can have a role in increasing median survival, which is 9.25 years in children with NF1 and 1.08 years in children without NF1. Barresi et al. found in their study that GBM with at least 30% giant cells is dominated by the impairment of the TP53/MDM3 and PTEN/PI3K pathways. They concluded that frequent RB1 alterations, hypermutation, and EGFR amplification occur in more aggressive cases [14]. The tumor cells in our study showed negative EGFR immunostain markers, which may predict a good prognostic factor in our case. Erson-Omay EZ et al. demonstrated that certain groups of GBM that harbored somatic in the exonuclease domain of the polymerase epsilon gene (POLE) mutations may show a good prognosis [15]. Our patient is a long-term free tumor survivor after 14 years of surgical and oncology management compared to the other cases of GBM described in the literature [2].

## Conclusions

Giant cell glioblastoma is a rare subtype of GBM with a better prognosis, especially if the patient was young and received multimodality management, and it is believed that associated NF1 can increase the chances of having a long and good survivor rate. As the tumor is very rare, we believe that we need more studies to know more about the causes behind the differences in prognosis in glioblastoma multiforme types.
